# *IGF2* mRNA Binding Protein 2 Transgenic Mice Are More Prone to Develop a Ductular Reaction and to Progress Toward Cirrhosis

**DOI:** 10.3389/fmed.2019.00179

**Published:** 2019-09-04

**Authors:** Beate Czepukojc, Ali Abuhaliema, Ahmad Barghash, Sascha Tierling, Norbert Naß, Yvette Simon, Christina Körbel, Cristina Cadenas, Noemi van Hul, Agapios Sachinidis, Jan G. Hengstler, Volkhard Helms, Matthias W. Laschke, Jörn Walter, Johannes Haybaeck, Isabelle Leclercq, Alexandra K. Kiemer, Sonja M. Kessler

**Affiliations:** ^1^Department of Pharmacy, Pharmaceutical Biology, Saarland University, Saarbrücken, Germany; ^2^Center for Bioinformatics, Saarland University, Saarbrücken, Germany; ^3^Department of Computer Science, German Jordanian University, Amman, Jordan; ^4^Genetics/Epigenetics, Saarland University, Saarbrücken, Germany; ^5^Department of Pathology, Medical Faculty, Otto von Guericke University Magdeburg, Magdeburg, Germany; ^6^Institute of Clinical-Experimental Surgery, Saarland University Hospital, Homburg, Germany; ^7^Systems Toxicology, Leibniz Research Centre for Working Environment and Human Factors (IfADo) at the TU Dortmund, Dortmund, Germany; ^8^Laboratory of Hepato-Gastroenterology, Institut de Recherche Expérimentale et Clinique, Université Catholique de Louvain, Brussels, Belgium; ^9^Department of Biosciences and Nutrition, Karolinska Institutet, Huddinge, Sweden; ^10^Center for Molecular Medicine Cologne (CMMC), Institute of Neurophysiology, University of Cologne, Cologne, Germany; ^11^Institute of Pathology, Medical University of Graz, Graz, Austria; ^12^Department of Pathology, Medical University Innsbruck, Innsbruck, Austria

**Keywords:** liver cancer, stem cell, de-differentiation, oval cell, HCC, fibrosis

## Abstract

The insulin-like growth factor 2 (*IGF2*) mRNA binding proteins (IMPs/IGF2BPs) IMP1 and 3 are regarded as oncofetal proteins, whereas the hepatic IMP2 expression in adults is controversially discussed. The splice variant IMP2-2/p62 promotes steatohepatitis and hepatocellular carcinoma. Aim of this study was to clarify whether IMP2 is expressed in the adult liver and influences progression toward cirrhosis. IMP2 was expressed at higher levels in embryonic compared to adult tissues as quantified in embryonic, newborn, and adult C57BL/6J mouse livers and suggested by analysis of publicly available human data. In an *IMP2-2* transgenic mouse model microarray and qPCR analyses revealed increased expression of liver progenitor cell (LPC) markers *Bex1, Prom1, Spp1*, and *Cdh1* indicating a de-differentiated liver cell phenotype. Induction of these LPC markers was confirmed in human cirrhotic tissue datasets. The LPC marker SPP1 has been described to play a major role in fibrogenesis. Thus, DNA methylation was investigated in order to decipher the regulatory mechanism of *Spp1* induction. In *IMP2-2* transgenic mouse livers single CpG sites were differentially methylated, as quantified by amplicon sequencing, whereas human HCC samples of a human publicly available dataset showed promoter hypomethylation. In order to study the impact of IMP2 on fibrogenesis in the context of steatohepatitis wild-type or *IMP2-2* transgenic mice were fed either a methionine-choline deficient (MCD) or a control diet for 2–12 weeks. MCD-fed *IMP2-2* transgenic mice showed a higher incidence of ductular reaction (DR), accompanied by hepatic stellate cell activation, extracellular matrix (ECM) deposition, and induction of the LPC markers *Spp1, Cdh1*, and *Afp* suggesting the occurrence of de-differentiated cells in transgenic livers. In human cirrhotic samples *IMP2* overexpression correlated with LPC marker and ECM component expression. Progression of liver disease was induced by combined MCD and diethylnitrosamine (DEN) treatment. Combined MCD-DEN treatment resulted in shorter survival of *IMP2-2* transgenic compared to wild-type mice. Only *IMP2-2* transgenic livers progressed to cirrhosis, which was accompanied by strong DR. In conclusion, IMP2 is an oncofetal protein in the liver that promotes DR characterized by de-differentiated cells toward steatohepatitis-associated cirrhosis development with poor survival.

## Introduction

The family of insulin-like growth factor (*IGF*) 2 mRNA binding proteins (IMPs/IGF2BPs), namely IMP1 and IMP3, have been described as oncofetal proteins. However, whether IMP2 is an oncofetal protein or not remains a matter of debate (1–3). IMP2 has been described to be essential for preserving glioblastoma cancer stem cells (4). It is known that the occurrence of hepatic stem cells, i.e., liver progenitor cells (LPCs) worsens prognosis in liver disease. Noteworthy, more than half of the earliest hepatic premalignant lesions consist of LPCs and intermediate hepatocytes (5). Human and murine HCCs show LPC gene signatures and contain LPC fractions (6). Concordantly, p53-deficient or H-RAS/SV40LT-transduced isolated LPCs can generate HCC in xenotransplant models (7, 8). In cholangiocarcinoma aberrant expression of stem cell markers suggest LPCs to be the cell of origin in tumors (9). Interestingly, mixed types of cholangiocarcinoma and HCC have been described in the literature, which are thought to derive from transformed progenitor cells (10). Thus, it has been suggested that LPCs may be precursor cells for malignant transformation (11). However, recent evidence using lineage tracing in different mouse models demonstrates that HCC rather seem to develop from dedifferentiated hepatocytes than from LPCs (12, 13).

Still, the majority of non-malignant chronic liver diseases are characterized by LPC activation (5). In ductular reaction (DR), activated LPCs organize in strings (14) and finally lead to increased numbers of bile ducts that form a denser mesh around portal vein branches (15) or even infiltrate into the lobular parenchyme (16). Interestingly, the degree of DR correlates with disease progression (17). DR comparable to the one found in human non-alcoholic steatohepatitis (NASH) can be induced by a methionine-choline deficient diet (MCD) showing high similarity with NASH samples with advanced fibrosis (18).

In this study, we confirm the oncofetal appearance of IMP2 in murine and human liver. Employing transgenic mice we show that IMP2-2 is able to promote DR resulting in cirrhosis and short survival in the steatohepatitis model. Human gene expression data further support the association of IMP2 with marker genes of undifferentiated or dedifferentiating cells in liver disease.

## Methods

### Animals

Liver tissue from C57BL/6J mice at embryonic day E12.5 (*n* = 5), newborns (P0, *n* = 8), and at the age of 2, 10, and 85 weeks (each *n* = 6) were obtained. Publicly available RNA sequencing data sets from murine (PRJNA66167) and human (PRJNA280600) fetal and adult liver (19) were analyzed.

Wildtype (wt) and *IMP2-2* transgenic (tg) mice were generated as previously described (20). At the age of 5 weeks, livers were examined by qPCR (wt: *n* = 13, tg: *n* = 13) and microarray analysis (wt: *n* = 10, tg: *n* = 10). In order to induce NASH, wt and *IMP2-2* tg mice were randomly divided into experimental groups at the age of 3 weeks and were fed either a methionine choline deficient diet (MCD, #960439, MP Biomedicals, Germany) or a MCD diet supplemented with choline bitartrate (2 g/kg) and DL-methionine (3 g/kg) (MCS, #960441, MP Biomedicals, Germany) for 2, 4, or 12 weeks (21) (2 weeks: MCS: wt: 5 females and 5 males; tg: 6 females and 4 males; MCD: wt: 5 females and 7 males; tg: 7 females and 5 males; 4 weeks: MCS: wt: 5 females and 5 males; tg: 5 females and 5 males; MCD: wt: 6 females and 6 males; tg: 6 females and 6 males; 12 weeks: MCS: wt: 5 females and 4 males; tg: 6 females and 5 males; MCD: wt: 5 females and 5 males; tg: 5 females and 4 males). For the combined treatment with MCD and the genotoxic carcinogen diethylnitrosamine (DEN) (22) two different treatment schemes were used: one set of 3 week old wt (*n* = 28; females: *n* = 14, males: *n* = 14) and *IMP2-2* tg (*n* = 24; females: *n* = 9, males: *n* = 15) mice received an MCD diet and 4 weeks after the onset of MCD feeding one injection of DEN (5 mg/kg BW i.p.). Mice were sacrificed at the age of 15 weeks or before when at least one of the stopping criterions (behavioral disorder, loss of weight >50%, abnormal water or feed uptake, matt coat, or an impairment of the locomotor system) was met. The other set of 2 week old wt (*n* = 10) and *IMP2-2* tg (*n* = 13) mice received a single injection of diethylnitrosamine (DEN, 5 mg/kg BW i.p.) and were fed a MCD diet at the age of 8 weeks (wt: *n* = 10, females: *n* = 5, males: *n* = 5; tg: *n* = 13, females: *n* = 7, males: *n* = 6). Mice were sacrificed at the age of 29 weeks. Mice were housed in a 12/12 h light/dark cycle under constant conditions (temperature: 22°C ± 2°C; relative humidity: 55% ± 10%) with an enriched environment and food and water *ad libitum*. Mice were sacrifized by cervical dislocation or decapitation (embros, newborns) and whole blood samples were taken and liver was excised. Two thirds of the liver was flash-frozen in liquid nitrogen and stored at −80°C for further analysis. One third of the liver containing half of the left lateral lobe and half of the medial lobe in the same cassette was fixed in 4% PBS-buffered formalin for 24 h. After fixation samples were dehydrated and embedded in paraffin.

Whole blood samples were incubated for 1 h at room temperature to allow agglutination of red blood cells and subsequently centrifuged for 10 min at 13,500 × g at 4°C. The supernatant was transferred into a fresh tube, diluted 1:3 with 0.9% NaCl, and stored at −80°C until measurement. Serum alanine aminotransferase (ALT), aspartate aminotransferase (AST), glucose, triglycerides (TG), cholesterol, and high density lipoprotein (HDL) levels were determined by a PPE Modular analyzer using Roche® reagents at a constant temperature of 37°C (Roche Diagnostics, Mannheim, Germany). Measurements were performed at the “Zentrallabor des Universitätsklinikums des Saarlandes” (Homburg, Germany).

### RNA Isolation, Reverse Transcription, and qPCR

In general, RNA isolation and qPCR were performed as previously described (23–25). Total RNA was extracted using Qiazol lysis reagent (Qiagen, Germany) according to the manufacturer's instructions and treated with DNase I (Ambion, Germany) to remove residual DNA. cDNA was synthesized using the high-capacity cDNA reverse transcription kit (Applied Biosystems) according to the manufacturer's protocol using random primers. qPCR was performed using 5x HOT FIREPol® EvaGreen® qPCR Mix Plus (Solis BioDyne, Estonia) according to the manufacturer's instructions and the CFX96 Touch™ Real-Time PCR Detection System (Bio-Rad, Germany). For absolute quantification PCR products were cloned into pGEMTeasy (Promega, Germany) and respective dilutions were run alongside the samples to generate a standard curve. All samples and standards were analyzed in triplicate using the following PCR conditions: 95°C for 15 min, followed by 39 cycles of 20 s at 94°C, 20 s at the primer-specific annealing temperature, and 20 s at 72°C. The relative expression was normalized to *18S* or *Ppia* mRNA values depending on their stability. In sum, three different housekeeping genes (18s, Ppia/Cyclophilin, and Csnk2a2) were tested using the GeNorm tool and Normfinder (26, 27). Real-time quantitative PCR (qRT-PCR) was performed using the following primer pairs: Rn18S 5′-AGGTCTGTGATGCCCTTA GA-3′ and 5′-GAATGGGGTTCAACGGGT TA-3′; Ppia 5′-GGCCGATGACGAGCCC-3′ and 5′-TGTCTTTGGAACTTTGTCTGC-3′; Afp 5′-CCAGGAAGTCTGTTTCACAGAAG-3′ and 5′-CAAAAGGCTCACACCAAAGAG-3′; Krt19 5′-AGCGTGATCAGCGGTTTTG-3′ and 5′-CCTGGTTCTGGCGCTCTATG-3′; Sox9 5′-CCAGCAAGAACAAGCCACAC-3′ and 5′-CTTGCCCAGAGTCTTGCTGA-3′; Spp1 5′-CCGAGGTGATAGCTTGGCTTAT-3′ and 5′-GACTCCTTAGACTCACCGCTC-3′; Cdh1 5′-CTTTTCGGAAGACTCCCGATT-3′ and 5′-GCTTTAGATGCCGCTTCACTGT-3′; Imp2 5′-CTGATCCCAGGGCTAAACCTC-3′ and 5′-AAGGGGTGATAGGGAGGACTG-3′; Dlk1 5′-ACTTGCGTGGACCTGGAGAA-3′ and 5′-CTGTTGGTTGCGGCTACGAT-3′.

### Western Blot Analysis

Western blots were performed as previously described (20).

### Histology

Histochemistry and immunohistochemistry were performed as previously described (21, 25, 28). Paraffin-embedded liver sections were stained with hematoxylin-eosin (HE) and Sirius Red. Immunohistochemical (IHC) KRT19 and AFP detection was achieved using Dako EnVision+ System-HRP Labeled Polymer Anti Rabbit (#K4003, DAKO, USA) with anti-KRT19 antibody (1:1,000, #52625, abcam, UK), anti-DLK1 (1:1,000, #21682, abcam, UK) or anti-AFP antibody (1:1,000, #46799, abcam, UK). Epitopes were demasked with citrate buffer (10 mM, pH 6.0) in a waterbath at 95°C for 45 min. For Laminin detection epitopes were demasked by proteinase K (20 μg/ml in TE buffer) incubation for 15 min at 37°C in a waterbath. Anti-laminin antibody (1:200, #L9393, Sigma) was incubated for 1 h at room temperature followed by incubation with anti-rabbit Envision (Dako, Germany) for 20 min at room temperature and DAB staining. Hyaluronic acid was detected by staining with a biotin-labeled hyaluronic acid binding protein (1:100, #AMS.HKD-BC41, Amsbio) for 1 h at room temperature and detection by Streptavidin-HRP (#K3954, Dako, Germany) for 20 min at room temperature and DAB staining. Stainings were evaluated for steatosis, inflammation, DR, and fibrosis by two independent, blinded investigators with the scoring system shown in Table 1. Fibrosis scoring was assessed according to Kleiner et al. (29). Steatosis scoring was performed according to Simon et al. (21). Scoring system for ductular reaction was based on Chen et al. (30). We adapted the scoring of the latter study by including two different cirrhosis scores: 3 = cirrhosis with few and 4 = cirrhosis with many DR (see Table 1). In order to avoid high complexity we additionally combined score 1 and 2 from Chen et al. into score 1 in the current study.

**Table 1:** Scoring system for histological analyses.

**Scoring system**			**Assessed by**
*Ductular reaction*	Score 0 Score 1 Score 2 Score 3 Score 4	None Rare-few (1–6 DR per field) Many (>6 DR per field) Cirrhosis with few DR Cirrhosis with many DR	HE and Krt19 IHC
*Fibrosis*	Score 0 Score 1 Score 2 Score 3 Score 4	None Perisinusoidal or periportal Perisinusoidal and portal/periportal and/or septal Bridging fibrosis portal/periportal and septal Cirrhosis (complete surrounding of liver cell nodules by fibrous septa)	Sirius red
*Steatosis*	Score 0 Score 1 Score 2 Score 3 Score 4	0% <5% >5–20% >20% >50%	HE
*Inflammation*	Portal Score 0 Score 1 Lobular Score 0 score 1 Score 2 score 3	None to minimal; greater than minimal; None <2/200x 2–4/200x >4/200x	HE

### Cell Culture

Huh7 cells were cultured in RPMI-1640 medium supplemented with fetal calf serum, penicillin/streptomycin and glutamine (Sigma-Aldrich, Taufkirchen, Germany) at 37°C and 5% CO_2_. Cells were treated with decitabine/5-aza-2′-deoxycytidine (#A3656, Sigma-Aldrich) over 3 days. The DNA-methyltransferase inhibitor was freshly added daily. Authentication of cell lines was confirmed by DSMZ.

### Amplicon Sequencing Analysis

Ten percent of each bisulfite-treated sample were used as template in a 30 μL PCR mix: buffer BD (80 mM Tris-HCL, 20 mM (NH_4_)_2_SO_4_), 2.5 mM MgCl_2_, 0.2 mM of each dNTP, 200 nm each of the forward and reverse primers (sequences see Table 2) with universal Illumina adapters attached at the 5′ end, and 3U HotFirePol (Solis BioDyne). For each DNA lot, PCR reactions were performed in triplicates starting with 15 min denaturation at 95°C followed by 42 cycles of 95°C for 60 s, annealing for 120 s (see Table 2), 72°C for 120 s, and a final 5-min extension step at 72°C. About 5 μL PCR reaction were loaded on 1.2% agarose gels. Remaining 25 μL of the PCR reaction were purified with Agencourt Ampure XP beads (Beckman Coulter, Krefeld, Germany), diluted, pooled, and sequenced (Illumina v3 chemistry, 2 × 300 bp paired end) on Illumina MiSeq following the manufacturer's instructions aiming at 10,000 reads per amplicon and sample. Alignments and evaluation were done with the BiQ Analyzer HT program filtering all reads with more than 10% unrecognized CpG sites (31). Primers used for PCR for amplicon sequencing of murine *Spp1* can be found in Table 2.

**Table 2:** Primers used for PCR for amplicon sequencing of murine *Spp1*.

**Ampli-con ID**	**Genomic position (mm10)**	**Primer forward (5′-3′)**	**Primer reverse (5′-3′)**	**Annealing temperature (°C)**
Spp1_1	chr5:104434413-104434742	GGTTATTGTGTGGTTTGTATAAAGAGT	CACATAAATACACAATTTAATTCTCTCAA	54
Spp1_2	chr5:104434987-104435392	TAAAATTAGAGGAGGAAGTGTAGGAGT	AAATTTATTTTAACTTCTACATCCAACTA	54
Spp1_3	chr5:104436033-104436270	TTTTTGTAGAAAGTATTTTTATGGTAGTT	TACATCTCAAAAAAAATTTCAATAAA	54
Spp1_4	chr5:104436350-104436768	TTGTGTTTTATAAAATATGTTGTAGGAT	AAAAAAATCTATTTATTAAAAACAATAAC	54
Spp1_5	chr5:104437141-104437598	ATTTTGTGTTTGGATAAAAAGGTATAT	AAAACTCTATAAACCATAACCTTAACATA	54
Spp1_6	chr5:104437693-104438080	GGTGTTTGATTTATTTTAGAAGTAGAATT	TATCCATTAATCCTCTAAACACTCAC	54
Spp1_7	chr5:104439171-104439512	AAGATTTTGAAATTTAATTAAAATGTTTT	AACCTCAATCCATAAACCAAACTAT	54
Spp1_8	chr5:104439713-104440076	TGTGTGTGATGTTTTTTTAATTTTATT	AATCAACTTCATATCAATCATTTCTAA	56
Spp1_9	chr5:104440214-104440610	TTGTTTTTGATTTAGTATTTTGATGTT	CCTCAAAAAATAAACTCTCTAATTCATA	60
Spp1_10	chr5:104440722-104441072	TGGATATATGTATGTAGAGAAAGAGAGGT	ATAATTAACACCACCAAACTCCTTACA	60

### Microarray Analysis

For Affymetrix gene expression microarray analysis RNA was extracted from snap frozen liver tissue of 5 week-old *IMP2-2* tg mice (*n* = 10) and corresponding controls (*n* = 10), as described in Godoy et al. (32). The Gene Chip IVT Labeling kit (Affymetrix, High Wycombe, UK) was used to generate biotinylated cRNA. Purification and fragmentation was performed according to Affymetrix's protocol. Hybridization to Mouse Genome 430 2.0 Affymetrix GeneChips (Santa Clara, CA, USA) was conducted for 16 h at 45°C according to the manufacturer's instructions. Microarrays were scanned with an Affymetrix scanner controlled by Affymetrix Microarray Suite software. Robust Multi-array Average (RMA) algorithm (33) was used for normalization of the gene expression array data and the R package limma (34) was used for calculation of differential gene expression. Adjustment for multiple testing was conducted with the method of Benjamini and Hochberg (FDR: false discovery rate) (35). Data are available on Gene Omnibus (GEO-ID: GSE130999).

### Public Gene Omnibus (GEO) and TCGA Datasets

GEO dataset GSE14323 and the liver hepatocellular carcinoma dataset from TCGA were analyzed as described previously (21, 25, 28, 36). The GEO dataset GSE14323 was analyzed for differential gene expression in human tissue samples of normal liver (*n* = 19), pre-malignant cirrhosis (*n* = 41), cirrhosis of HCC-livers (*n* = 17), and HCC (*n* = 48) as described previously (21, 25, 28, 36). TCGA liver cancer samples (*n* = 159) were analyzed for promoter methylation of *SPP1*. Statistical significance was analyzed in the R-cran statistical environment by the Kolmogorov–Smirnov test followed by FDR-correction with the Benjamini-Hochberg method *Q* = 0.05, and up to *m* = 10 (35). Pearson correlation was applied to detect correlations between genes of interest.

### High-Resolution Ultrasound

Mice were anesthesized with isoflurane and put on a heated stage in supine position. Chemical depilation (Nair hair removal cream) was done to prevent air trapped in the fur from interfering with ultrasound coupling into the animal. After removing the liver, ultrasound coupling gel (Aquasonic 100, Parker, Fairfield, NJ, USA) was circumferentially applied to the *ex situ* liver, to distinguish between the stage and the *ex situ* liver. Imaging was performed by means of a Vevo 770 high-resolution ultrasound system (VisualSonics, Toronto, ON, Canada) and a real-time microvisualization 704 Scanhead (VisualSonics) with a center frequency of 40 MHz and a focal depth of 6 mm. For 3D imaging the scanhead, driven by a linear motor, scanned first the *in situ* and then the *ex situ* liver. The technique yielded two-dimensional (2D) images at parallel and uniformly spaced 100 μm steps. The 2D image planes enabled rapid 3D image reconstruction, displaying a dynamic cube view format, as previously described (37).

### Statistical Analysis

OriginPro 2018G software (OriginLab Corporation) was used for illustration and statistical analyses. Results are shown as means ± SEM or box plots with 25th/75th percentile boxes, geometric medians (line), means (square). Statistical significance was determined by Mann-Whitney *U*-test or *t*-test where applicable. Chi^2^ test was used for categorical data. Statistics of Kaplan-Meier survival analysis was performed by log rank test.

## Results

### Resource Identification Initiative

**Figure 1:**
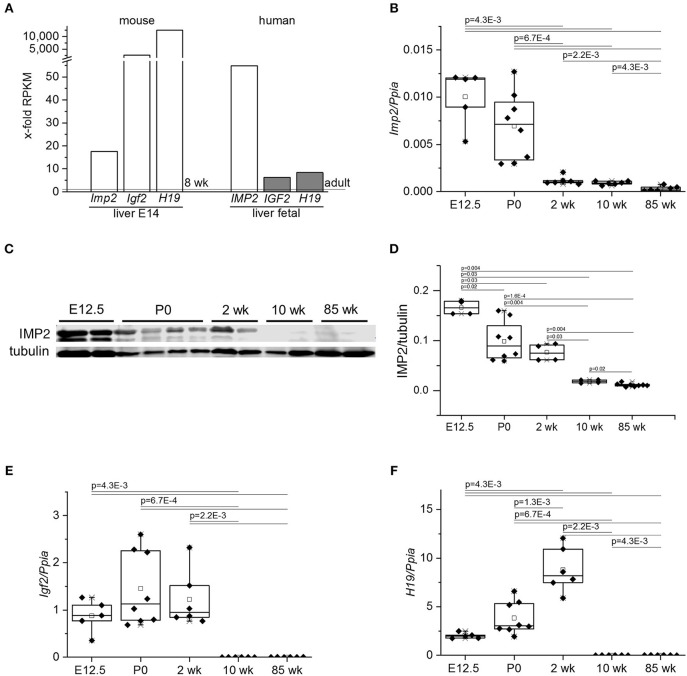
Expression of *IMP2, IGF2*, and *H19* in embryonic and adult livers. **(A)** *IMP2, IGF2*, and *H19* gene expression from publicly available RNA sequencing data sets of murine (PRJNA66167) and human (PRJNA280600) fetal liver compared to adults. Data are expressed as x-fold from 8 week-old murine or adult human tissues indicated by dashed lines, respectively. RPKM: Reads per kilobase transcript per million reads. **(B,E,F)** qPCR analysis of *Imp2* **(B)**, *Igf2* **(E)**, and *H19* **(F)** in livers of fetal (E12.5; *n* = 5), newborns (P0, *n* = 8), 2, 10, and 85 week-old (each *n* = 6) mice. Expression levels were normalized to *Ppia*/*Cyclophilin* as housekeeping gene. **(C,D)** Western blot analysis of IMP2 protein expression in fetal, newborn, young, and adult mouse livers. Representative image of Western blot **(C)** and densitometric quantification of IMP2 levels normalized to tubulin **(D)** are shown (E12.5: *n* = 4; P0: *n* = 8; 2 and 10 week: *n* = 4; 85 week: *n* = 6). *P*-values were calculated by Mann-Whitney *U*-test.

Analysis of publicly available gene expression data showed high *IMP2* expression in fetal mouse and human livers compared to the respective adult tissue (Figure 1A). Embryonic markers, such as *IGF2* and *H19*, were highly expressed in fetal livers (Figure 1A). The low expression of *Imp2* in adult and high expression in fetal liver was confirmed by qPCR analysis of a time course of fetal, neonatal, young, and old mice (Figure 1B). Expression of IMP2 protein matched that of its transcript (Figures 1C,D). Further, the high levels of *H19* and *Igf2* in embryonic livers and low levels in adult livers were confirmed in the same time course (Figures 1E,F). Interestingly, *H19* expression was highest in livers of newborn and young animals (Figure 1F).

**Figure 2:**
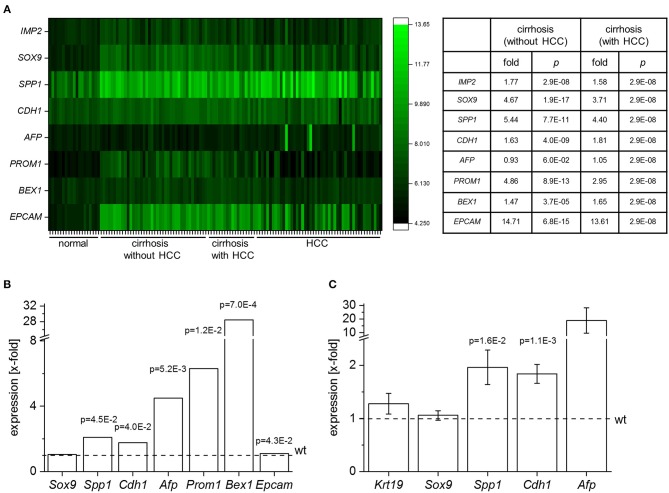
IMP2-2 induces stem cell marker expression. **(A)** Expression analysis of *IMP2, SOX9, SPP1, CDH1, AFP, PROM1, BEX1, EPCAM* in human samples of normal liver tissue (*n* = 19), cirrhotic tissue (without HCC; *n* = 41), cirrhotic tissue of HCC-livers (with HCC; *n* = 17), and HCC tissue (*n* = 48) (GSE14323). The table shows expression values as x-fold of healthy (*n* = 19) liver samples. *P*-values were generated by comparison to healthy tissue (Mann Whitney *U*-test or Welch's *t*-test, FDR-corrected by Benjamini-Hochberg method). **(B)** Gene expression of *Sox9, Spp1, Cdh1, Afp, Prom1, Bex1*, and *Epcam* in 5 week-old *IMP2-2* transgenic animals (tg) determined by micro array analysis (*n* = 10 per genotype). Data are expressed as x-fold of wildtype (wt) animals (indicated as dashed line). FDR adjusted p-values are shown. For *Afp* median value of five different probes is shown (probe 1: x-fold = 17.62, adj. *p* = 0.002; probe 2: x-fold = 5.35, adj. *p* = 0.008; probe 3: x-fold = 4.52, adj. *p* = 0.005; probe 4: x-fold = 1.38, adj. *p* = 0.009; probe 5: x-fold = 0.95, adj. *p* = 0.35). **(C)** *Krt19, Sox9, Spp1, Cdh1*, and *Afp* mRNA expression of *IMP2-2* tg mice (*n* = 13) normalized to the values of wt mice (*n* = 13). *P*-values were calculated in comparison with wt animals (Welch's *t*-test, FDR-corrected by Benjamini-Hochberg method).

These data showed the highest IMP2 expression in embryonic liver. We therefore hypothesized that IMP2 in adult liver may represent a marker of immaturity or dedifferentiation and aimed at investigating the impact of *IMP2* expression on hepatic progenitor cell features. Since peritumoral DR correlates with fibrosis stage (38) we first analyzed the expression of *IMP2* and common progenitor cell marker genes in gene expression data sets from human cirrhotic tissue (Figure 2A). Human cirrhotic and cirrhosis-associated HCC tissues showed an increase of *IMP2* expression and of the progenitor cell markers *SOX9, SPP1/osteopontin, CDH1/E-cadherin, AFP, PROM1*/*CD133, BEX1, EPCAM* (Figure 2A). We next investigated the consequences of overexpressing *IMP2-2* in the liver. Array analysis of *IMP2-2* tg mice revealed an upregulation of the murine homologs LPC marker genes except for *Sox9* (Figure 2B). Upregulation was confirmed by qPCR for some of the genes, i.e., *Spp1, Afp*, and *Cdh1* (Figure 2C). *Krt19*, which was not on the array platform and *Sox9*, both markers of DR, were not upregulated (Figure 2C).

**Figure 3:**
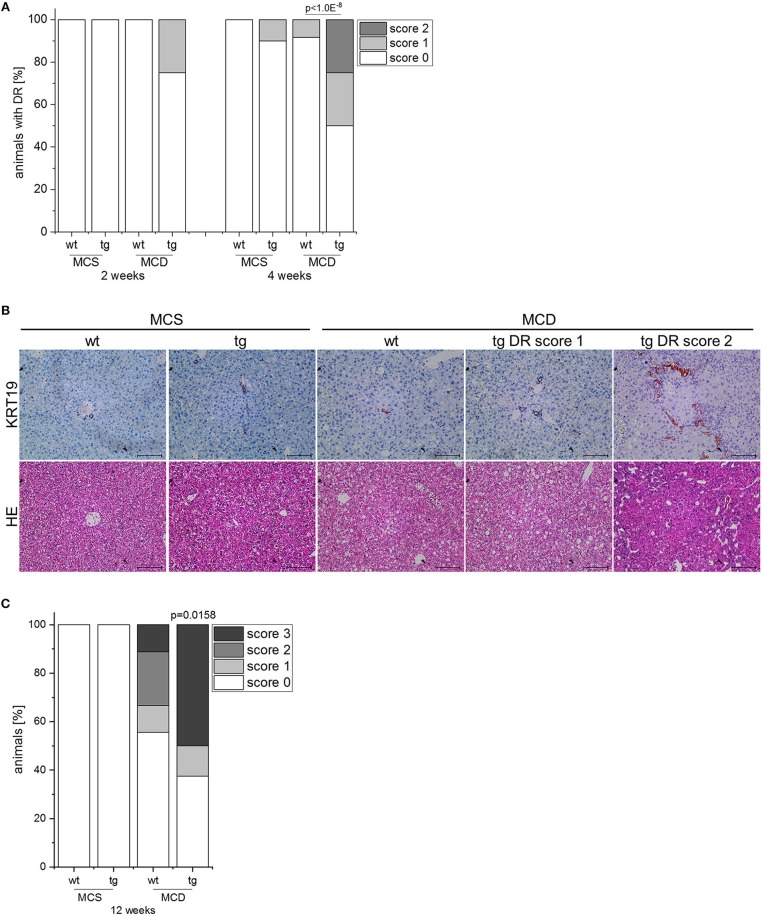
IMP2-2 expression accelerates DR in the MCD model. **(A)** Histological scoring of DR in *IMP2-2* transgenic (tg) and wildtype (wt) mice, which were fed either a methionine-choline deficient (MCD) diet or a methionine-choline supplemented (MCS) control diet for 2 and 4 weeks (2 weeks: MCS: *n* = 10 per genotype, MCD: *n* = 12 per genotype; 4 weeks: *n* = 10 per genotype, MCD: *n* = 12 per genotype). The *p*-value was calculated by chi-square test. **(B)** Representative images of HE and KRT19 stainings from wt and tg mice on MCS or MCD diet for 4 weeks. For tg mice representative images of DR score 1 and DR score 2 are shown (scale bars: 100 μm). **(C)** Histological scoring of Sirius red stained sections for fibrosis of wt and tg mice, which were fed either a methionine-choline deficient (MCD) diet or a methionine-choline supplemented (MCS) control diet for 12 weeks (MCS: *n* = 9–11 per genotype, MCD: *n* = 9–10 per genotype).

**Figure 4:**
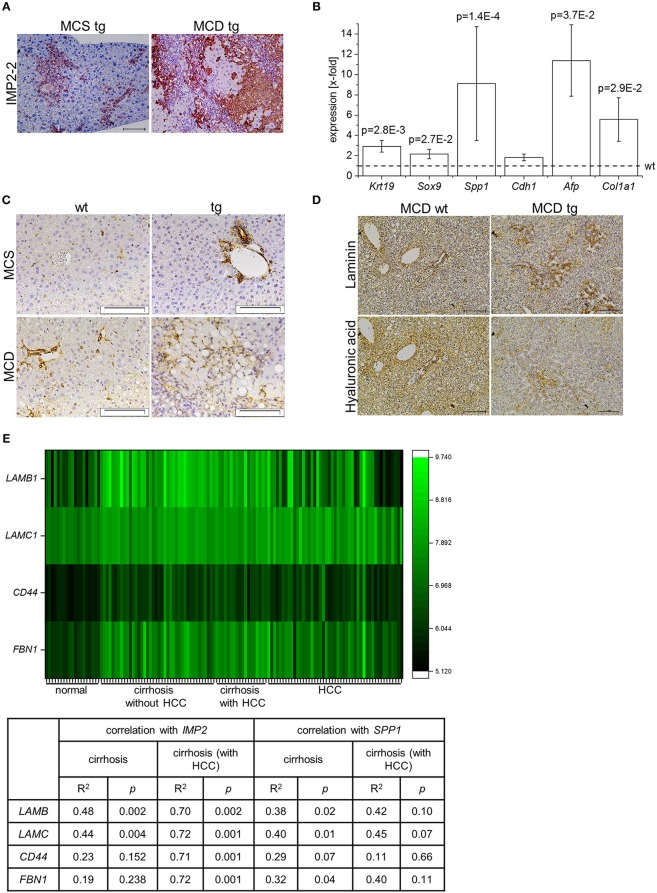
*IMP2*-2 expression correlates with the expression of extracellular matrix components. **(A)** Representative images of IMP2 staining in tg mice on MCS or MCD diet for 4 weeks (scale bars: 100 μm). **(B)** *Krt19, Sox9, Spp1, Cdh1, Afp, Col1a1* mRNA expression levels of 4 weeks MCD-fed IMP2-2 tg mice (*n* = 12), shown as x-fold of wild-types (*n* = 12; indicated by dashed line). *18S* was used as a housekeeping gene. Statistical significance compared to wt was determined by Mann-Whitney *U*-test or Welch's *t*-test. **(C)** Representative immunohistochemical staining for α-SMA in wt and tg mice fed a MCD or a MCS control diet for 4 weeks (MCS: *n* = 10 per genotype, MCD: *n* = 12 per genotype; scale bars: 50 μm). **(D)** Representative image of an area of DR stained for laminin (upper panel) and hyaluronic acid (lower panel) in wt and tg mice fed a MCD diet for 4 weeks (*n* = 12 per genotype; scale bars: 100 μm). **(E)** Heatmap shows expression of *LAMB1, LAMC1, CD44* and *FBN1* in human samples of normal liver tissue (*n* = 19), cirrhotic tissue (*n* = 41), cirrhotic tissue of HCC-livers (*n* = 17), and HCC tissue (*n* = 48) (GSE14323). The table shows a correlation analysis of *IMP2* and *SPP1* with *LAMB1, LAMC1, CD44*, and *FBN1* in human samples of cirrhotic tissue (*n* = 41) and cirrhotic tissue of HCC-livers (*n* = 17) (GSE14323). The correlation coefficient was calculated by Pearson correlation.

*IMP2-2* tg mice have been shown to develop steatosis without any sign of liver damage (20). Due to the increased levels of markers of de-differentiation, *IMP2-2* tg mice were challenged with an MCD diet, which is known to induce DR within 8 weeks in the setting of steatohepatitis (18). In *IMP2-2* tg mice DR was observed in 25% of the *IMP2-2* tg animals fed the MCD diet already after 2 weeks (Figure 3A). On the control diet (MCS) none of the *IMP2-2* tg mice of the 2 week time point and only 10% of the *IMP2-2* tg mice of the 4 week timepoint developed DR (Figure 3A). More pronounced DR was observed in the *IMP2-2* tg livers after 4 weeks of MCD feeding as shown by immunohistochemistry of KRT19 (Figures 3A,B). After 12 weeks of MCD diet more severe DR was observed in wt as well as in *IMP2-2* tg mice with no statistically significant difference between both genotypes (data not shown), but fibrosis was still increased in the transgenics (Figure 3C). Interestingly, the *IMP2-2* transgene was not only expressed in hepatocytes but also DR cells under MCD feeding (Figure 4A). After 4 weeks DR in *IMP2-2* tg mice was characterized by increased expression of the cholangiocyte and progenitor cell markers *Krt19* and *Sox9* (Figure 4B). The progenitor cell marker genes *Spp1* and *Afp* were induced to a higher extent in MCD-fed *IMP2-2* tg mice compared to wt livers (Figure 4B). *Cdh1* tended to be increased in *IMP2-2* tg livers on MCD diet (Figure 4B). *Igf2* levels were especially increased in female mice (data not shown), while the increase of the other progenitor cell markers in tg mice were sex independent. Since SPP1 was shown to activate hepatic stellate cells (HSCs), which can then produce extracellular matrix (ECM) components, we investigated α-smooth muscle actin (α-SMA) and *Col1a1* levels as a measure of differentiation to myofibroblasts in MCD-fed mice and observed increased *Col1a1* mRNA levels and α-SMA positive cells in *IMP2-2* tg animals (Figures 4B,C). Myofibroblasts are known to produce ECM components. Thus, immunohistochemistry was performed to investigate the deposition of ECM components in the areas of DR of *IMP2-2* tg livers. DR cells were surrounded by the ECM components laminin and hyaluronic acid (Figure 4D). In human cirrhotic tissue *SPP1* was upregulated (Figure 2A) and strongly correlated with *IMP2* expression (*R*^2^ = 0.42 *p* = 0.006 in cirrhosis). Further correlation analysis revealed a strong association of *IMP2* and *SPP1* with ECM components, i.e., laminin b (*LAMB*), laminin c (*LAMC*), hyaluronic acid receptor *CD44*, and fibrillin 1 (*FBN1*) (Figure 4E).

**Figure 5:**
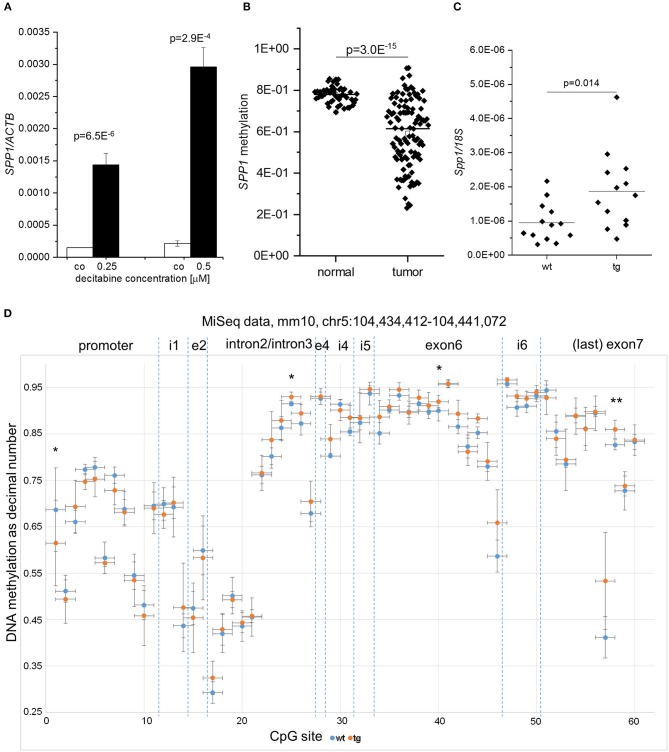
Differential *SPP1* methylation in human HCC and *IMP2-2* tg mice. **(A)** *SPP1* expression in Huh7 cells treated with either 0.25 or 0.5 μM decitabine for 72 h quantified by qPCR. Decitabine-containing medium was changed every 24 h. **(B)** *SPP1* promoter methylation in the TCGA liver cancer set in tumor vs. non-tumorous tissues. **(C)** *Spp1* expression in *IMP2-2* tg and wt mice on normal chow quantified by qPCR. *18S* was used as housekeeping gene. **(D)** Methylation of CpG sites within the *Spp1* gene in *IMP2-2* tg (orange) vs. wt (blue) mice analyzed by amplicon sequencing. CpG sites are plotted along the x-axis according to their location in the gene, i.e., in the promoter region, introns [intron1 (i1), intron2/intron3, intron4 (i4), intron5 (i5), and intron6 (i6)], and exons (exon2 (e2), exon4 (e4), exon6, exon7). ^*^p < 0.05; ^**^p < 0.01.

SPP1 is known to promote fibrogenesis (39) thereby linking liver progenitor cell activation to fibrotic changes (40). Since *Spp1* expression was reported to be regulated by altered DNA methylation in murine CCl_4_-induced fibrosis (41), we hypothesized that *SPP1* hypomethylation might cause *SPP1* induction in HCC. Therefore, we measured *SPP1* expression after incubation of HCC cells with the demethylating agent decitabine as well as *SPP1* DNA methylation in human samples. In fact, decitabine treatment in *in vitro* cultivated HCC cells strongly induced *SPP1* expression (Figure 5A). *In silico* analysis of the human TCGA HCC dataset revealed *SPP1* promoter hypomethylation in HCC patient tissues compared to normal liver tissues (Figure 5B). In order to test whether elevated *Spp1* expression in untreated *IMP2-2* tg mice is due to epigenetic changes we performed amplicon-based local deep sequencing covering the whole *Spp1* gene with 60 single CpG positions in *IMP2-2* tg and wt mice (42). Although *Spp1* expression was significantly elevated in untreated *IMP2-2* tg mice (Figure 5C), only minor methylation changes in single CpG sites of *Spp1* were observed (Figure 5D). CpG#1 was significantly hypomethylated in *IMP2-2* tg compared to wt mice (Figure 5D).

**Figure 6:**
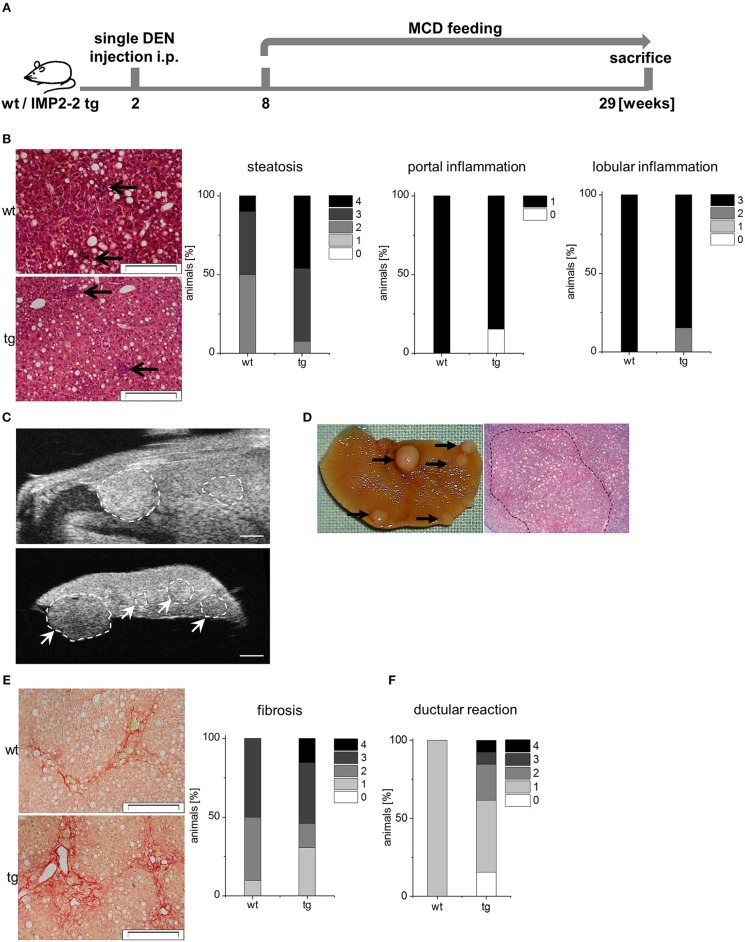
IMP2-2 triggers cirrhotic changes in the DEN-MCD model. **(A)** Treatment scheme of the DEN/MCD model. Administration of diethylnitrosamine (DEN) 6 weeks before the onset of MCD feeding (DEN-MCD). **(B)** Representative images of HE (left) and histological scorings for steatosis, lobular and portal inflammation of wt and tg mice treated with DEN-MCD. Arrows indicate inflammatory foci (scale bars: 50 μm). **(C,D)** Representative images of high-resolution ultrasound **(C)** and macroscopic view **(D)** of a liver of DEN-MCD mice. **(C)** High-resolution ultrasound imaging of an *in situ* (upper image) and *ex situ* (lower image) liver. Scale bars 0.9 mm (upper image), 1.2 mm (lower image). **(E)** Representative images of Sirius Red staining (left) and histological scoring for fibrosis (right) of wt and tg mice treated with DEN-MCD (wt: *n* = 10, tg: *n* = 13) (wt: score 2; tg: score 4; scale bars: 50 μm). **(F)** Histological scoring of KRT19 staining for DR (right) of wt and tg mice treated with DEN-MCD (wt: *n* = 10, tg: *n* = 13).

**Figure 7:**
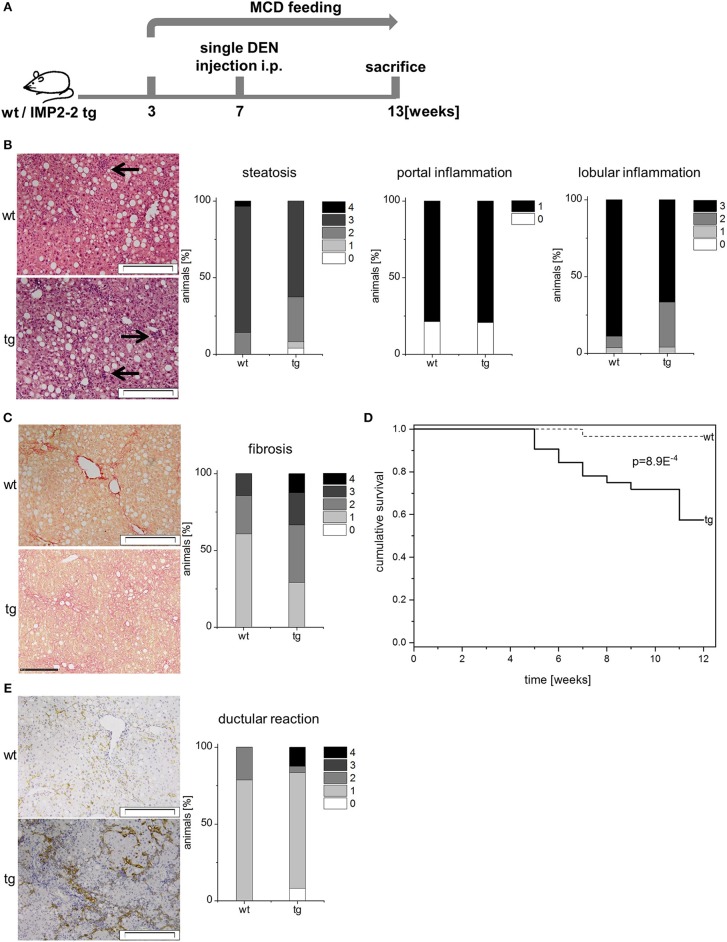
Transgenic expression of IMP2-2 leads to shorter survival in the MCD-DEN model. **(A)** Treatment scheme of the MCD/DEN model. Administration of DEN 5 weeks after the onset of MCD feeding (MCD-DEN). **(B)** Representative images of HE (left) and histological scorings for steatosis, lobular and portal inflammation of wt and tg mice treated with MCD-DEN (wt: *n* = 28, tg: *n* = 24). Arrows indicate inflammatory foci (scale bars: 50 μm). **(C)** Representative images of Sirius Red staining (left) and histological scoring for fibrosis (right) of wt and tg mice treated with MCD-DEN (wt: *n* = 28, tg: *n* = 24) (wt: score 1; tg: score 3; scale bars: 50 μm). **(D)** Kaplan-Meier survival plot referring to wt and tg animals treated with MCD-DEN (wt: *n* = 28, tg: *n* = 24). **(E)** Representative images of KRT19 staining (left) and histological scoring for DR (right) of wt and tg mice treated with MCD-DEN (wt: *n* = 28, tg: *n* = 24) (scale bars: 200 μm).

Since DR associates with fibrosis, it is associated with poor prognosis in liver disease (43). Therefore, we analyzed whether additional administration of the genotoxic carcinogen DEN caused more severe fibrosis in *IMP2-2* tg livers. Two different treatment schemes were used with DEN injection before or after the onset of MCD feeding (Figures 6A, 7A).

In the DEN-MCD treated animals (Figure 6A) high ALT and AST levels were found (Table 3). No difference between the genotypes regarding AST, ALT, triglycerides, cholesterol, HDL, and glucose were observed (Table 3). Steatosis ranged from score 2–4 in both genotypes and also inflammation was rather advanced in both genotypes (Figure 6B). Ultrasound analysis followed by macroscopic analysis exhibited the presence of cirrhotic nodules in *IMP2-2* tg livers (nodule size >0.5 mm: wt: *n* = 2 with and *n* = 8 without nodules, tg: *n* = 10 with and *n* = 1 without nodules, *p* = 0.0019 tg compared to wt; Figures 6C,D). Sirius red staining confirmed fibrotic changes leading to cirrhosis only in the *IMP2-2* tg livers (Figure 6E) with a varying extent of DR in the transgenics, while wt livers only showed weak DR (Figure 6F).

**Table 3:** Serum parameters in DEN-MCD and MCD-DEN treated mice.

	**DEN-MCD**		**MCD-DEN**	
	**wt**	**tg**	**wt**	**tg**
ALT [U/l]	409.7 ± 129.6	428.7 ± 151.6	80.6 ± 15.5	60.3 ± 13.5
AST [U/l]	3,550.9 ± 1,122.9	2,580.3 ± 912.3	2,901.1 ± 558.3	2,970.6 ± 664.2
Triglycerides [mg/dl]	96.2 ± 30.4	82.4 ± 29.1	127.5 ± 24.5	131.2 ± 29.3
Cholesterol [mg/dl]	35.0 ± 11.1	33.0 ± 11.7	48.3 ± 9.3	47.9 ± 10.7
HDL [mg/dl]	17.6 ± 5.6	18.4 ± 5.8	6.7 ± 1.3	5.4 ± 1.2
Glucose [mg/dl]	55.6 ± 17.6	57.3 ± 20.2	40.1 ± 7.7	44.1 ± 9.8

*Means ± SEM are shown*.

The MCD-DEN model (Figure 7A) revealed no difference in serum parameters (AST, ALT, triglycerides, cholesterin, HDL, and glucose), steatosis, and inflammation between the genotypes (Figure 7A; Table 3). In line with the other treatment scheme no difference between the genotypes was observed regarding steatosis and inflammation (Figure 7B), but cirrhosis was only present in the *IMP2-2* tg livers (Figure 7C) with only few macroscopically visible nodules with no significant difference between wt and tg regarding occurrence of nodules. In the MCD-DEN set cirrhosis was linked to a significantly shorter survival time of the transgenic animals compared to wt controls (*p* = 0.0056, Figure 7D). The MCD-DEN treatment group contained livers showing cirrhosis accompanied by strong DR (Figure 7E). Investigation of two progenitor cell markers, DLK1 and AFP, revealed a significant upregulation of *Afp* mRNA and protein levels in the *IMP2-2* tg animals in the MCD-DEN model (Supplementary Figures 1, 2).

## Discussion

IMPs were originally identified and characterized as fetal proteins (44). Although IMP2 was first described as an oncofetal protein (3), *IMP2* mRNA was suggested to be ubiquitously expressed in adult tissue (1). However, our data clearly show that IMP2 RNA and protein is expressed in fetal but not in adult human and mouse liver. IMP2 has been described to be re-expressed in liver cancer (45) and to preserve glioblastoma stem cells (4) underlining its oncofetal character. Moreover, *IMP2* is more abundant than *IMP1* and *IMP3* in most cancer types and promotes an undifferentiated character of HCC (28).

Due to this oncofetal expression in liver we hypothesized that IMP2-2 might affect liver pathogenesis related to a de-differentiated cell phenotype. We show that *IMP2-2* tg livers were more susceptible to DR provoked by an MCD diet. In untreated livers expression of the *IMP2-2* transgene did not result in DR but caused elevated expression of progenitor cell markers.

In untreated livers of *IMP2-2* tg mice a complex picture was obtained, characterized by the upregulated biliary/progenitor cell markers (*Spp1*), fetal hepatocytic marker (*Afp*), and classical stem/progenitor cell markers (*Cdh1, Prom1, Bex1*, and *Epcam*). Challenging these mice with the MCD diet resulted in DR and additional upregulation of biliary/progenitor cell markers (*Krt19* and *Sox9*). In general, DR, which mostly constitutes of cells of immature biliary or hepatobiliary phenotype, are diverse in their cellular components (46). Although SPP1 and SOX9 usually represent cholangiocyte-like cell markers (47, 48), they have been shown to be also expressed in de-differentiated mature hepatocytes upon on injury by 3,5-diethoxycarbonyl-1,4-dihydrocollidine (DDC) (49). Furthermore, a de-differentiated SPP1/SOX9-positive hepatocyte population has been described in DDC-injured livers, which can be found near Epcam-positive expanding DR cells (50). Upregulation of *Afp* in the IMP2-2 livers (51) further suggests a de-differentiation process (52). Moreover, AFP expression was associated with EPCAM and PROM1 expression in BEX1-acclerated liver injury in the choline-deficient, ethionine-supplemented (CDE) diet model (53).

Interestingly, *IMP2-2* tg mice showed significantly elevated levels of *Cdh1*, which is an epithelial marker overexpressed in LPCs. CDH1 expression can enhance maintenance of embryonic stem cell pluripotency [reviewed in (54)] and progressively increases with elevated numbers of progenitor cells (55). CDH1 expressing cells probably overlap with cells positive for DLK1 (56), which is also induced in *IMP2-2* tg livers (28). In the fetal liver, DLK1 is specific for AFP-positive hepatoblasts (57). As the biliary markers *Krt19* and *Sox9* were not altered in *IMP2-2* tg mice on normal chow, *IMP2-2* tg mice seem to have an increased amount of de-differentiated hepatocyte-like cells (58). Still, as DLK1-positive progenitor cells are bipotential progenitor cells (59), they might also be the source of the accelerated occurrence of KRT19-positive DR cells observed on MCD feeding in *IMP2-2* tg animals with significantly increased levels of both biliary markers *Sox9* and *Krt19*. After partial hepatectomy proliferation of a specific subpopulation of cells positive for KRT19, AFP, and DLK1 has been described (58). Taken together, the MCD diet seems to provoke DR in *IMP2-2* tg mice, which is characterized by a mixed cell population of a de-differentiated or undifferentiated phenotype.

Expression of SPP1 is associated with severe NASH in humans (60). *SPP1* expression has been shown to be regulated by promoter methylation in liver fibrosis (41). Methylation changes of the *SPP1* gene differed between human HCC and our *IMP2-2* mouse model. Only single CpG sites were altered regarding methylation. CpG#1, which was hypomethylated in the *IMP2-2* tg mice, is located within the binding site of runt-related transcription factor 2 (Runx2) in the *Spp1* promoter. Interestingly, reduced methylation of CpG#1 of the human *SPP1* promoter was reported to influence transcription factor binding in SH-SY5Y bone marrow cells, thereby leading to increased *SPP1* expression (61) suggesting a similar mechanism for IMP2-2 induced *Spp1* expression. SPP1 is able to activate HSCs leading to ECM production (62, 63) thereby promoting fibrosis. Immunohistochemical stainings of *IMP2-2* tg livers of MCD-fed mice revealed a morphologically mixed type of DR cells in small nests and in bile duct-like formations. More precisely, ECM deposition and activation of matrix-producing cells occurred in front of proliferating DR cells as it has been described for the CDE model (55). Also progenitor cells positive for DLK1 were reported to be surrounded by a laminin layer in the rat liver (58). The laminin and hyaluronic acid staining pattern in *IMP2-2* tg livers supports the hypothesis that the DR cell compartments are surrounded and supported by an ECM layer (55).

The shortened survival in *IMP2-2* tg mice in MCD-DEN regime shows that IMP2-2 increases liver damage in chronic liver disease. The *IMP2-2* transgenic mice were more susceptible toward DR and fibrosis caused by the MCD diet alone, since DR was already observed after 2 weeks of feeding. The difference between the genotypes was even more pronounced after 4 weeks, but became similar between *IMP2-2* transgenic and wt mice after 12 weeks. This is explained by the fact that 12 weeks of MCD diet are generally accepted to be sufficient for induction of DR and fibrosis in wild-type mice (18). Therefore, the increased susceptibility of the *IMP2-2* tg mice is probably masked by the advanced diet-induced liver disease in the latest analyzed time point of 12 weeks. With an additional challenge, such as DEN, *IMP2-2* tg livers were again more susceptible. In fact, cirrhosis only occurred in *IMP2-2* tg livers, which is probably due to the triggered progression of fibrosis by IMP2-2 (21).

Taken together, hepatic *IMP2-2* expression leads to a de-differentiated cell population and enhanced DR, which promotes the development of cirrhosis in the MCD mouse model. Concordantly, also human cirrhotic samples expressed high *IMP2* levels suggesting a similar role for IMP2 in human cirrhosis.

## Ethics Statement

This study was carried out in accordance with the recommendations of the German guideline TierSchG and the European guideline 2010/63/EU. The protocol was approved by the animal welfare committee of Saarland University (permission number: 34/2010, 11/2013).

## Author Contributions

BC, AA, AB, ST, NN, YS, CK, CC, NH, and SK conceived and carried out experiments and analyzed the data. AS, JGH, VH, ML, JW, JH, IL, and AK analyzed the data. SK concepted and supervised the study. All authors were involved in writing the paper and had final approval of the submitted and published versions.

### Conflict of Interest Statement

The authors declare that the research was conducted in the absence of any commercial or financial relationships that could be construed as a potential conflict of interest.
