# Supplementary material for: IGF2 mRNA Binding Protein 2 Transgenic Mice Are More Prone to Develop a Ductular Reaction and to Progress Toward Cirrhosis
Source: Front Med (Lausanne). 2019 Sep 4;6:179. doi: 10.3389/fmed.2019.00179 (PMC6737005; doi:10.3389/fmed.2019.00179)
Supplement: Supplementary file 1 [file Data_Sheet_1.pdf]

Supplementary data

IGF2 mRNA binding protein 2 transgenic mice are more prone to develop a ductular reaction and to progress-towards cirrhosis

Beate Czepukojc[1], Ali Abuhaliema[1], Ahmad Barghash[2,3], Sascha Tierling[4], Norbert Naß[5], Yvette Simon[1], Christina Körbel[6], Cristina Cadenas[7], Noemi van Hul[8,9], Agapios Sachinidis[10], Jan G. Hengstler[7], Volkhard Helms[2], Matthias W. Laschke[6], Jörn Walter[4], Johannes Haybaeck[5,11,12], Isabelle Leclercq[8], Alexandra K. Kiemer[1], Sonja M. Kessler[1,8,11*]

[1] Department of Pharmacy, Pharmaceutical Biology, Saarland University, Saarbrücken, Germany

[2] Center for Bioinformatics, Saarland University, Saarbrücken, Germany

[3] Department of Computer Science, German Jordanian University, Amman, Jordan

[4] Genetics/Epigenetics, Saarland University, Saarbrücken, Germany

[5] Department of Pathology, Medical Faculty, Otto von Guericke University Magdeburg, Magdeburg, Germany

[6] Institute of Clinical-Experimental Surgery, Saarland University Hospital, Homburg, Germany

[7] Systems Toxicology, Leibniz Research Centre for Working Environment and Human Factors (IfADo) at the TU Dortmund, Dortmund, Germany

[8] Laboratory of Hepato-Gastroenterology, Institut de Recherche Expérimentale et Clinique, Université Catholique de Louvain, Brussels, Belgium

[9] Department of Biosciences and Nutrition, Karolinska Institutet, Huddinge, Sweden

[10] Center for Molecular Medicine Cologne (CMMC), Institute of Neurophysiology, University of Cologne, Cologne, Germany

[11] Institute of Pathology, Medical University of Graz, Graz, Austria

[12] Department of Pathology, Medical University Innsbruck, Innsbruck, Austria

***Correspondence:**
Sonja M. Kessler
s.kessler@mx.uni-saarland.de

Table of contents

Supplementary Figures

Figure S1 3

Figure S2 4

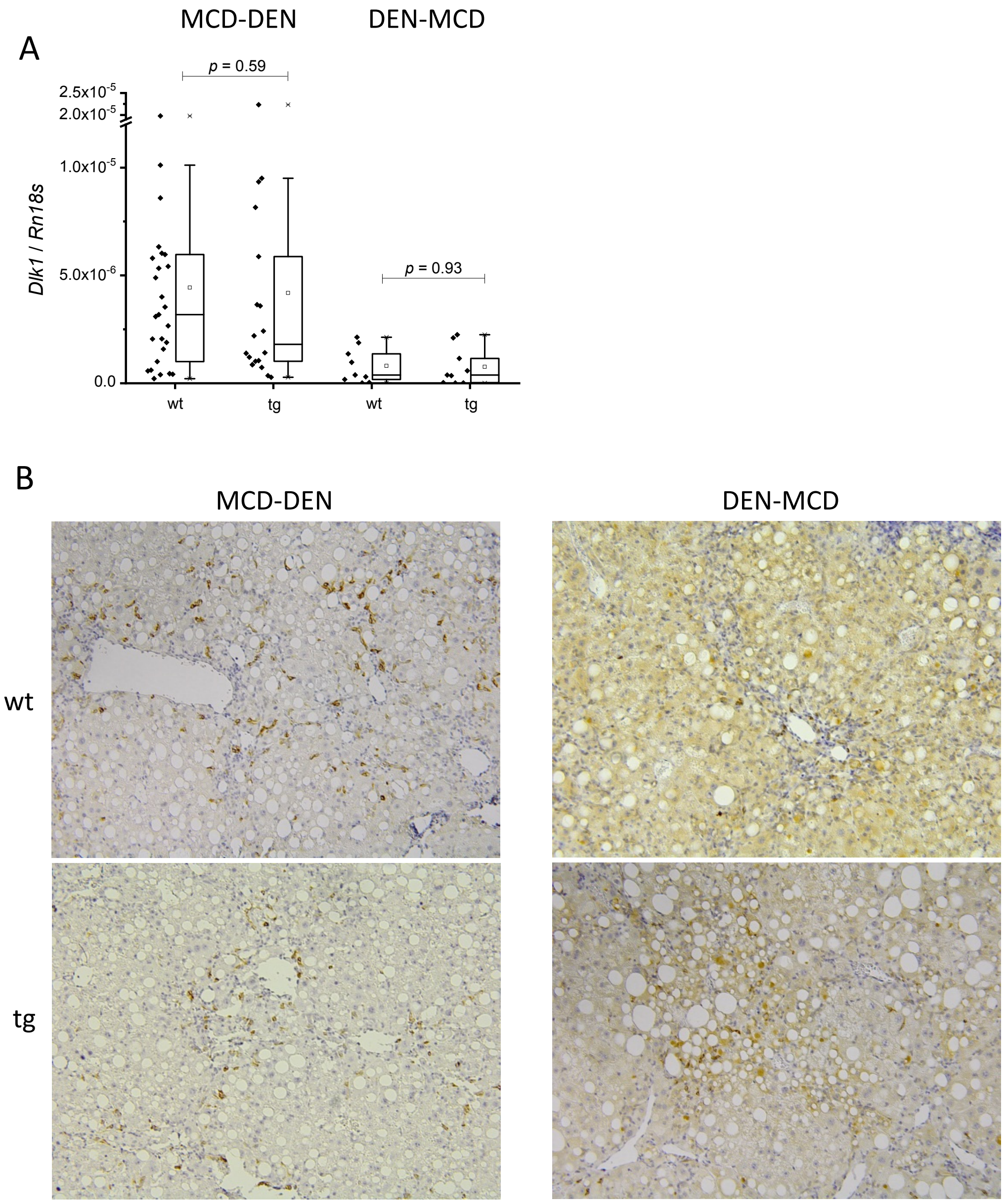

Fig. S1: ***Dlk1*** **expression in the DEN-MCD and the MCD-DEN models. (A)** ***Dlk1*** **gene expression in** ***IMP2-2*** **tg and wt mice treated with MCD-DEN or DEN-MCD, respectively, quantified by qPCR. 18S was used as housekeeping gene. Data are shown as single values and box plots with median (–) und mean (□). (B) Representative images of DLK1 staining of wt and tg mice treated with DEN-MCD or MCD-DEN, respectively (original magnification 200 x). DLK1 stainings were categorized in four scores: no staining, weak staining, medium staining, and strong staining. In the MCD-DEN model tissues exhibited the following scores: no staining: wt: 14.3%, tg: 20.0%; weak staining: wt: 42.9%, tg: 56.0%, medium staining: wt: 17.9%, tg: 16.0%; strong staining: wt: 25.0%, tg: 8.0%. In the DEN-MCD model the following scores were observed: no staining: wt: 16.7%, tg: 8.3%; weak staining: wt: 75.0%, tg: 83.3%, medium staining: wt: 8.3%, tg: 8.3%; strong staining: wt and tg: 0% each.**

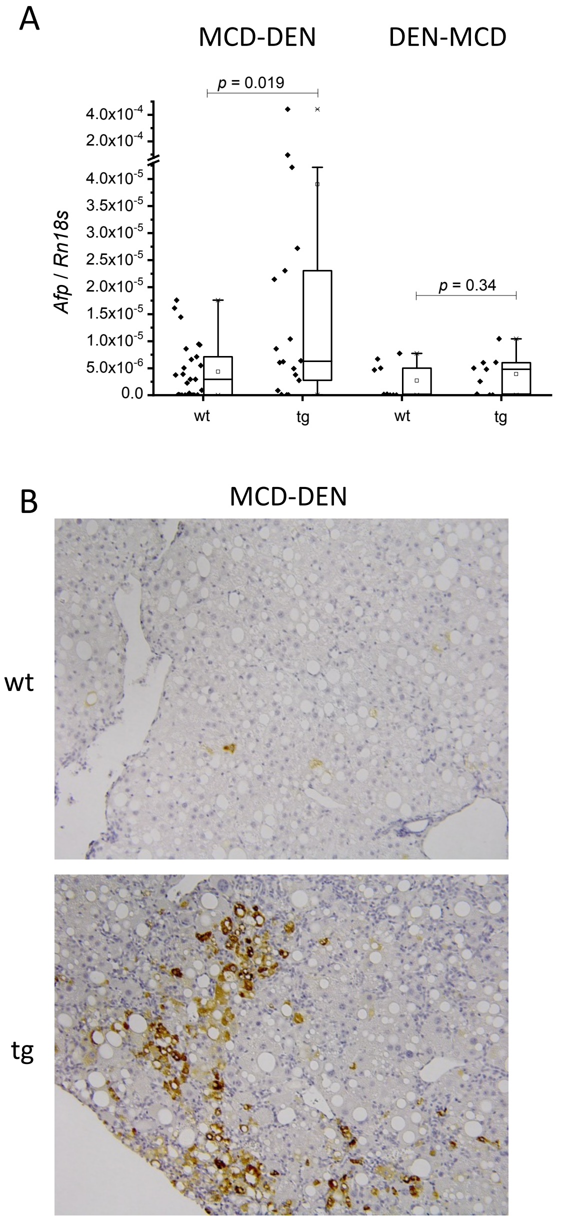

Fig. S2: Afp expression in the DEN-MCD and the MCD-DEN models. (A) *Afp* gene expression in *IMP2-2* tg and wt mice treated with MCD-DEN or DEN-MCD, respectively, quantified by qPCR. 18S was used as housekeeping gene. Data are shown as single values and box plots with median (–) und mean (□). (B) Representative images of AFP staining of wt and tg mice treated with MCD-DEN, respectively (original magnification 200 x). AFP stainings were categorized in four scores: no staining, weak staining, medium staining, and strong staining. In the MCD-DEN model tissues exhibited the following scores: no staining: wt: 14.8%, tg: 0%; weak staining: wt: 55.6%, tg: 13.6%, medium staining: wt: 25.9%, tg: 40.9%; strong staining: wt: 3.7%, tg: 45.5% (p=0.00064 Chi^2-test).
